# The Knowledge of Eye Physicians on Local Anesthetic Toxicity and Intravenous Lipid Treatment: Questionnaire Study

**DOI:** 10.4274/tjo.79446

**Published:** 2017-12-25

**Authors:** Aykut Urfalıoğlu, Selma Urfalıoğlu, Gözen Öksüz

**Affiliations:** 1 Sütçü İmam University Faculty of Medicine, Department of Anesthesiology and Reanimation, Kahramanmaraş, Turkey; 2 Necip Fazıl State Hospital, Opthalmology Clinic, Kahramanmaraş, Turkey

**Keywords:** Ophthalmologist, local anesthesia toxicity syndrome, intravenous lipid solution

## Abstract

**Objectives:**

To evaluate the knowledge of ophthalmologists regarding local anesthesia toxicity syndrome (LATS) and intravenous lipid emulsion used in treatment, and to raise awareness of this issue.

**Materials and Methods:**

A questionnaire comprising 14 questions about demographics, local anesthesia (LA) use, toxicity, and treatment methods was administered to ophthalmologists at different hospitals.

**Results:**

The study included 104 ophthalmologists (25% residents, 67.3% specialists, 7.7% faculty members) with a mean age of 35.71±6.53 years. The highest number of participants was from state hospitals (65.4%), and 34.6% of the physicians had been working in ophthalmology for more than 10 years. Seventy-six percent of the participants reported using LA every day or more than twice a week, but 56.7% had received no specific training on this subject. No statistically significant difference was observed between different education levels and the rates of training (p=0.419). Bupivacaine was the most preferred LA and the majority of respondents (97.1%) did not use a test dose. Allergy (76%) and hypotension (68.3%) were the most common responses for early findings of LATS, while cardiac arrest (57.4%) and hepatotoxicity (56.4%) were given for late findings. The most common responses concerning the prevention of LATS included monitorization (72.4%) and use of appropriate doses (58.2%). Symptomatic treatment was selected by 72.4% of respondents and cardiopulmonary resuscitation and antihistamine treatment by 58.8%. Of the ophthalmologists in the study, 62.5% had never encountered LATS. The use of 20% intravenous lipid emulsion therapy for toxicity was known by 65% of the physicians, but only 1 participant stated having used it previously.

**Conclusion:**

The importance of using 20% lipid emulsion in LATS treatment and having it available where LA is administered must be emphasized, and there should be compulsory training programs for ophthalmologists on this subject.

## INTRODUCTION

Local anesthetics enable surgical procedures to be performed without requiring general anesthesia, but can result in a number of complications related both to the patient being awake and to administration errors. One of these complications is local anesthesia toxicity syndrome (LATS). Although rare, it can result in death if not treated early. LATS in the central nervous system (CNS) may initially manifest with non-specific findings such as a metallic taste in the mouth, perioral numbness, tinnitus, general malaise, slurred speech, and diplopia. However, these early signs are not always present; symptoms may begin with CNS excitation (agitation, confusion, convulsions) and progress if not treated to findings of depression (mental depression, coma, apnea). Cardiovascular system (CVS) findings may occur simultaneously with CNS signs or appear later, and can include hyperdynamic findings such as hypertension and tachyarrhythmia as well as signs of cardiac depression such as hypotension, bradyarrhythmia, conduction block, and asystole.^1^

The first guideline to facilitate the early recognition and treatment of LATS was published by the Association of Anaesthetists of Great Britain and Ireland in 2007 and was revised in 2010.^2^ The common theme emphasized both in this guideline and later by the American Regional Anesthesia Society in 2010 and 2012 and the American Academy of Clinical Toxicologists in 2015 is the use of 20% intravenous lipid emulsion (IVLE) in combination with supportive therapy for the treatment of LATS.^3,4,5^ In particular, it is known that cardiac arrest in LATS is resistant to standard resuscitation methods, and that IVLE therapy is critical as an initial therapy.^6^ Although the mechanism of action is not fully known, lipids have been shown to exert a scavenging effect by binding local anesthetics in the circulation, and a direct inotropic effect by improving mitochondrial function of cardiac cells and increasing calcium uptake.1 They have also been shown to provide significant symptomatic improvement in patients at various LATS stages with and without cardiac arrest.^7,8,9^

The majority of ophthalmic procedures (e.g., eyelid, cataract, strabismus, keratoplasty, and vitreoretinal surgeries) are performed under local anesthesia.^10^ LATS can be recognized early and controlled in most cases with proper operating room monitorization and support from an anesthesia team. However, the true problem lies in the fact that most of these procedures are conducted in local operating rooms remote from well-equipped surgical theaters, without adequate monitoring or anesthesiologists in attendance. Therefore, it is important for ophthalmologists who frequently use local anesthesia to be aware of the early and late symptoms of LATS and to implement appropriate treatment options promptly when necessary.

In our literature review, we found studies applying similar surveys in all branches using local anesthetics, but there were no studies conducted exclusively among ophthalmologists. Considering the widespread use of local anesthesia in ophthalmology practice, the aim of this study was to increase awareness of this issue by evaluating the knowledge of ophthalmologists at all stages of training regarding local anesthetic toxicity and intravenous lipid emulsion used in its treatment.

## MATERIALS AND METHODS

The study was approved by the Sütçü İmam University Faculty of Medicine Clinical Research Ethics Committee (2017/02-02). The purpose and nature of the study were explained to all physicians and verbal consent was obtained from all participants prior to the study. A total of 104 ophthalmologists employed in different positions at different hospitals were asked to respond to a questionnaire consisting of 14 items concerning demographic information, their local anesthetic use, toxicity, and treatment methods. The questionnaire was applied in person when possible; otherwise, responses were collected by telephone or e-mail. The questionnaire was adapted from questions used in previous studies by Başaranoğlu et al.^11^ and Karasu et al.^12^

### Statistical Analysis

Statistical analyses were done using IBM SPSS for Windows, version 22.0 (IBM Corporation, Armonk, New York, USA). Numerical variables are expressed as mean ± standard deviation, categorical variables as number and percentage. P<0.05 was accepted as the level of significance.

## RESULTS

**Figure 1:**
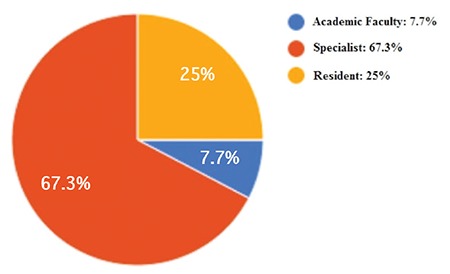
Education level of the surveyed ophthalmologists

A total of 104 ophthalmologists participated in the survey and all provided appropriate responses to all of the questions. The mean age of the participants was 35.71±6.53 years; 25% were residents, 67.3% were specialists, and 7.7% were academic faculty members (Figure 1). There were more participants from state hospitals (65.4%) than from university and private hospitals. In terms of professional experience, participants practicing ophthalmology for 10 or more years comprised the largest subgroup, with 34.6%. Seventy-six percent of the participants used local anesthetics every day or more than twice a week, though 56.7% of them stated that they had not received any training in the use of local anesthetics during their education. There were no statistically significant differences in education received about local anesthetics based on the participants’ education level or years of experience in ophthalmology (p=0.419). Bupivacaine was the most preferred local anesthetic among the physicians (61%). Despite the known cardiotoxic effects of bupivacaine, 97.1% of the participants reported not using a test dose prior to local anesthetic administration. The demographic data and information regarding local anesthetic use of the surveyed physicians are shown in Table 1.

The participants’ responses concerning local anesthetic toxicity and its treatment are given in Table 2. Of all the participants, 62.5% had never encountered local anesthetic toxicity. Allergy (76%) and hypotension (68.3%) were associated with early findings of toxicity, while cardiac arrest (57.4%) and hepatotoxicity (56.4%) were given as late findings. When asked how toxicity could be prevented, 72.4% said monitorization and 58.2% said by administering appropriate doses. Regarding what treatment is necessary in the event of toxicity, 72.4% of the participants said symptomatic therapy, and 58.8% said cardiopulmonary resuscitation and antihistaminics. Sixty-five percent of the participants had never heard of 20% IVLE therapy in toxicity; 3.9% stated that they knew this treatment was used in toxicity, but only 0.96% of the participants reported previously using 20% IVLE in the treatment of toxicity.

## DISCUSSION

This survey study demonstrated that there is some general knowledge about local anesthesia and LATS among ophthalmologists. However, despite the frequent use of local anesthetic agents in this branch of medicine, the education practitioners receive about this topic is insufficient. In particular, our results indicate that the majority of ophthalmologists are also not adequately informed about the use of lipid emulsions, which have been shown to effectively treat toxicity and are advised to have on hand wherever local anesthesia is practiced.

The ability to perform most ocular surgeries under local anesthesia is a great advantage in terms of avoiding complications associated with general anesthesia in this patient group, who are usually older adults with comorbid conditions. Despite the recent development of topical eye drop anesthesia to reduce complication rates, most ophthalmologists prefer injection anesthesia because it provides faster and stronger anesthesia as well as a more comfortable surgery due to akinesia.^10^ Despite all of these advantages, injection anesthesia methods such as peribulbar anesthesia, sub-Tenon’s block, and especially retrobulbar anesthesia can also lead to several complications.^13,14,15^ Allergic reactions to the local anesthetic agents, hypoglycemia, stroke, oculocardiac reflex, and the potentially fatal LATS are among these complications.^14^ Although LATS occurs rarely, it may lead to fatal outcomes if early intervention is not provided due to lack of awareness or the appropriate therapy cannot be given.^1^ The high preference for local anesthesia makes it imperative that ophthalmologists receive continuing education concerning local anesthetics and LATS. Although 76% of the participants reported using local anesthetics every day or more than twice a week, 56.7% had not received any training in the use of local anesthetics during their education.

The characteristics of the local anesthetic agent used are also important in the development of LATS. Because ophthalmic anesthesia is generally applied in small amounts, toxic overdose is very rare and the characteristics of the local anesthetic agent are more relevant. Bupivacaine is an agent with serious cardiotoxic potential and patients who go into cardiac arrest due to bupivacaine-induced LATS are known to be resistant to resuscitation.^1^ Our questionnaire revealed that bupivacaine was most preferred by the participants, likely due to its longer duration of action compared to other agents, but hardly any of the physicians used test doses. Due to the lack of adequate training on local anesthetics, the cardiotoxic effects of bupivacaine were not well known, suggesting that its long-acting nature was the sole reason for its popularity.

Besides the characteristics of the local anesthetic used, two main mechanisms related to the means of administration have been implicated in the development of LATS in ocular surgeries. The first mechanism is that the drug is accidentally injected into the ophthalmic artery, resulting in retrograde spread to the internal carotid artery and then the brain. In the second mechanism, the dural sheath surrounding the optic nerve is accidentally punctured, resulting in spread of the drug to the brain via the subdural and subarachnoid space. These can occur due to not performing aspiration prior to intraarterial injections, and using a long needle or not ensuring the eye is in neutral position for intrameningeal injections. Therefore, in addition to appropriate monitoring, is it recommended that aspiration be done before every injection, that the eye be in neutral position during injection, that shorter needles be used, and that methods such as ultrasound be used to help determine the injection site when applying local anesthesia.^16^ Regarding how to prevent LATS, the participants in our study gave more priority to monitorization and use of appropriate doses of local anesthetics rather than methods such as aspiration test and intermittent injection which indicate improper intra-arterial injection technique.

Our results indicated that a majority (62.5%) of the participants had never encountered LATS; they most commonly listed allergies and hypotension as early signs and cardiac arrest and hepatotoxicity as late signs. Although the findings of LATS are generally classified as early and late findings, the clinical presentation may not always follow this order. In most cases, CNS involvement first appears with non-specific findings such as a metallic taste in the mouth, perioral numbness, tinnitus, lightheadedness, and slurred speech; however, it may manifest with convulsions and progress to coma and respiratory depression. CVS manifestations can exhibit a wide spectrum at every stage, ranging from signs of stimulation (hypertension, tachyarrhythmia) to depression (hypotension, bradyarrhythmia, cardiac arrest).^1^ In ophthalmic anesthesia, more importance is given to onset time and the mechanisms by which toxicity occurs, rather than to the sequence of LATS findings. In particular, apnea or cardiac manifestations occur within seconds with intraarterial injections, while these findings appear more slowly with intrameningeal spread, over the course of minutes.^16^ A large study of retrobulbar block including 6,000 patients showed that symptoms appeared after 2-40 min (mean 8 min) due to probable meningeal spread of local anesthetic to the CNS.13 In contrast, Dettoraki et al.^17^ reported a case receiving retrobulbar block for vitrectomy in which convulsions and contralateral hemiparesis occurred immediately after local anesthetic was administered due to intraarterial injection.

In addition to closely following patients receiving local anesthesia with appropriate monitoring and intravenous catheterization, guidelines released in recent years have emphasized the importance of airway control, 100% O_2_ ventilation, anticonvulsive therapy, and the use of 20% IVLE with resuscitation in case of cardiac arrest in patients who develop LATS.^2,4^ When toxicity is suspected, it is recommended to initiate 20% IVLE with a bolus dose of 1.5 mL/kg and continue with infusion at 15 mL/kg/hr. If symptoms do not improve, two additional bolus doses can be administered and therapy continued to a maximum dose of 10 mL/kg.^1^ Our literature search yielded no studies concerning intralipid therapy for patients with LATS associated with ophthalmic anesthesia, but rapid improvement of LATS symptoms has been demonstrated with 20% IVLE therapy in other surgical settings, both in patients with and without cardiac arrest.^7^ In a study conducted by Başaranoğlu et al.^11^ among physicians who frequently use local anesthesia, it was found that 65.7% of physicians in all branches had never heard of this treatment in relation to LATS and 21.4% said they could not recall, whereas 70.4% of anesthetists were aware of lipid therapy. Similarly, a survey of residents from all branches conducted by Karasu et al.^12^ revealed that 67.4% of the participants had never heard of this treatment. In addition, despite a high rate of training among anesthesiology residents (76.9%), some of the other clinical residents reported having had no training on this subject. This high rate among anesthesiologists may be attributable to the frequent use of peripheral and central blocks. Nevertheless, a study conducted among anesthetists in Denmark showed that although 65% were aware of the use of lipid therapy to treat LATS, only 8 (24%) anesthetists knew the treatment protocol and only 1 (3%) of the anesthetists had ever witnessed the use of lipid in the clinic.^18^ In the present study, 20% IVLE therapy was generally unrecognized by ophthalmologists as a treatment for LATS. This may be explained by the fact that they had never heard of this treatment or, even if they had, were not adequately trained in this subject, or by the low frequency of LATS encounters.

### Study Limitations

In this survey, we collected responses to the prepared questionnaire via face-to-face interviews with the ophthalmologists we were able to reach and via phone and e-mail for the others. Although we contacted as many physicians as we could within a certain time, the participation rate was not very high. For this reason, we think that conducting such surveys through established associations in the relevant field would yield higher participation rates.

## CONCLUSION

LATS is rare but can be fatal if intervention is delayed. The inclusion of this subject in the compulsory curriculum would significantly increase awareness in ophthalmology practices, where local anesthesia is frequently used. In particular, the importance of 20% IVLE therapy in LATS treatment and the need for 20% lipid emulsion to be available to ophthalmologists wherever they apply local anesthesia should be emphasized.

## Figures and Tables

**Table 1:**
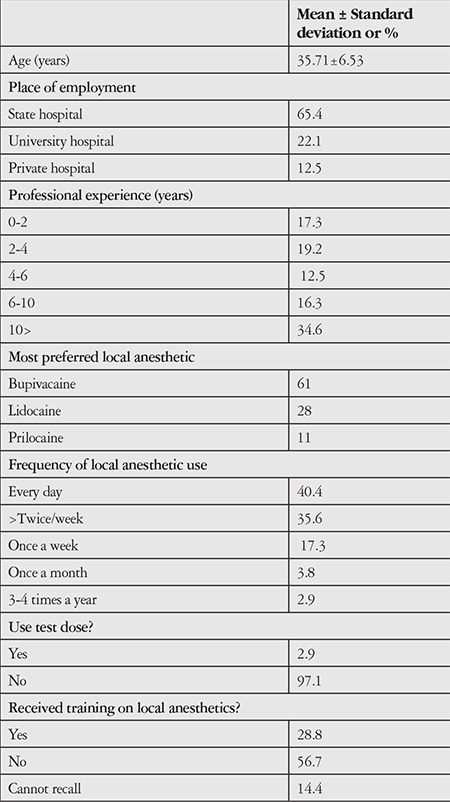
Demographic data of the survey participants and their responses regarding local anesthetic use

**Table 2:**
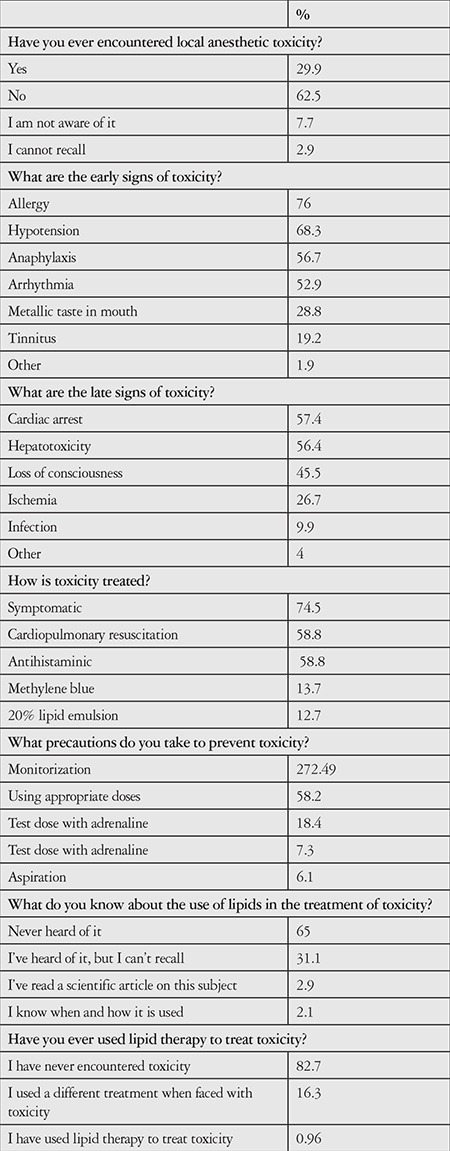
The participants’ responses concerning local anesthetic toxicity and its treatment
